# A Statistical Report of the Walcheren Fever, as It Appeared among the Troops at Ipswich, on Their Return from Holland

**Published:** 1811-01

**Authors:** W. Hamilton

**Affiliations:** Ipswich


### Medical and Physical Journal.

VOL. XXV.] JANUARY, 1811. [NO. 143.

Printed for R. PHILLIPS, by E. Hemsted, Great New Street, Fetter Lane, London.

*To the Editors of the Medical and Physical Journal.*

### *A statistical Report of the* WALCHEREN FEVER, *as it appeared among the troops at Ipswich, on their return from Holland.*

GENTLEMEN,

THE first of the sick arrived at Ipswich on the 10th of September, 1809, several of whom were found, when removed from the waggons, in a comatose stupid state, in which they continued till death. The greater number, however, were afflicted with intermittents and remittents of irregular and anomalous types, often united with pulmonic and bowel complaints. Almost every week, and sometimes daily, men were brought to the Hospitals and Barrack rooms, the latter of which were now converted into Wards, for the convenience of the sick. The number increased to between five and six hundred. Those who recovered were always discharged, as soon as possible, to provide for the worst cases among the fresh arrivals.

Of the intermittents, which were the chief and original complaints, very few died affected with this disease alone. When they terminated fatally the disorder, some days previous to death, first changed into a continued fever, and afterwards assumed the typhoid type, with great pain of the head, anorexia, tongue covered with a brown crust, low mutterings and delirium. When reduced to this state, few escaped. Those who recovered relapsed so frequently, that many were discharged and re-admitted in the course of the same week. This return appeared after the slightest change of weather or exposure to cold, many falling down while on parade, and others during the time they stood on guard. To avoid this as much as possible, all those who were discharged from the hospital were placed in convalescent barracks, and excused every kind of duty for several weeks, and when thus seasoned were discharged. The relapses, however, were found to be as numerous among these convalescents, as among those who were at duty; and it was remarked, that the men who had recovered in Walcheren were less liable to relapses than the others who recovered at home. Perhaps this may be accounted for, by the duration of the disease inducing greater debility. In fact, the men continued in such a weak, languid state till the beginning of June 1810, that few were able to bear the least excitement of whatever kind, without causing relapses. These returns of the disease were also frequently united with pulmonic complaints, which increasing soon terminated fatally. In these critical cases all the efforts of the medical practitioner were too often of no avail, for while he endeavoured to evade Scylla, he was sure to fall on Charybdis.

The time this disease lay latent in some habits, who were not affected with it while in Holland, appears a curious fact; several being attacked with different species of intermittents after they had been home seven or eight months. Many officers as well as privates were thus attacked at different subsequent periods.

As an example of the extent to which this malignant endemic proceeded, I need only mention, that out of one battalion consisting of about seven hundred men, only 21 escaped its attack, and about an hundred of the others fell its victims.

About the middle of October a great many were attacked with hydropic complaints, so that one third almost of the patients who laboured under the different forms of intermittents became dropsical. At first hydrothorax and universal anasarca often suddenly appeared, which increased by the violence of the paroxysms of ague, frequently terminated fatally during the exacerbation. I was present at three of these afflicting scenes, even conversing with my patient a few minutes before his exit, which was so unexpected, that I saw one rise out of bed and place himself on the close stool, where he expired in a few seconds. Another robust, athletic man was seized with a violent paroxysm of ague, when his respiration became so difficult and hurried, that he was obliged to sit up in bed to assist his breathing. His thirst was insatiable. He spoke frequently, and said he should die; and with these words he leaned himself backwards and immediately expired. Being present I took hold of his hand, when I felt the pulse beat, which continued its pulsations for near two minutes after respiration and other vital appearances ceased. This man had no other external appearance of dropsy than that of a slight tumefaction about his ancles. On examining amining the body a large quantity of water was found in the thorax, and near six ounces in the pericardium. The abdomen also contained some serous fluid.

The third case was a man who had just arrived, and who appeared so well that he was sent to the convalescent Wards where I saw him, at which time no hydropic complaint appeared. An universal anasarca attacked him so quickly that he died a few hours after. On viewing the body the skin seemed so distended on every part, that it was ready to burst, and a general exudation had commenced, especially on the scrotum and thighs.

These dropsical affections had now become so prevalent, that it was a general rule particularly to examine the patient concerning them.*

Some time afterwards Dysenteria appeared, and the sick increasing, it very soon became a frequent and fatal malady. The disease was, in general, preceded by a diarrhæa. The sick labouring under the greatest debility from the length of time they had been afflicted, and from the immense quantities of bark and other medicines administered, among which purgatives held a principal station, it will not be difficult to conceive what must be the consequence. The bowels thus repeatedly harassed and irritated with these medicines, gradually lost their tone, when diarrhæa was produced; and as the intermittents often did not even then subside, it was kept up by the continued exhibition of the remedies for the original complaint.

When therefore the purging was not checked by proper remedies, it soon degenerated into a dysentery, which was peculiarly distinguished by that most harassing symptom tenesmus, accompanied with bloody mucous stools, and continued pain of the hypogastric region; for the support of which flannel rollers were constantly used, and found highly beneficial. This disease being often combined with others was seldom without fever. Many died soon after its appearance, so that some medical men concluded that no man who was attacked with it would recover. This conclusion, however, proved to be false. Several languished for many weeks, till they became so emaciated as to appear nothing more than skin and bones (to use the vulgar phrase) and at length recovered. When it appeared in this chronic state, the patient's appetite remained good till death, which generally snatched him off during his slumbers.

* Boerhaave mentions this disease as frequently occurring in Holland, particularly among robust people, to whom, after much muscular action, cold and inactivity soon succeed.

As there were many unexpected deaths from the hydropic complaints, so there were several very unexpected recoveries from this. Of this I could relate many examples. This recovery, however, appeared the more extraordinary, when we compared the dreadful ravages the disease had made on those who died of it. On inspecting their bodies, the larger intestines were almost always inflamed, thickened, covered with ulcers, and in many places sphacelating.

Icterus very frequently appeared as a symptom of this disease, but was scarcely ever observed as an original complaint. It even appeared in one instance, where the patient was undergoing a slight salivation, and what was most remarkable, there was no appearance of any bilious effusion until the mercury began to act. It also subsided in a ratio corresponding with the disappearance of the salivation. This case shews more fully the peculiar action of mercury on the liver.

Another very troublesome symptom of this disease was ardor urinæ. Many were so much afflicted with this complaint, as to discharge frequently a quantity of blood either immediately before or after micturition. Large round worms from six to twelve inches long were often voided by vomiting. These had often induced the most distressing symptoms.

These were the most common forms of this Proteus-malady, for a more particular account of which, I must refer to Dr. Davis's Treatise, and shall now beg leave to proceed with a cursory view of the means which were used to counteract its progress.

### TREATMENT OF THE WALCHEREN FEVER.

As the disease appeared under so many different forms, the treatment was, of course, various, for which reason I shall follow the three forms mentioned above.

### *Intermittents and Remittents, &c.*

In these complaints it was usual first to exhibit a cathartic, and afterwards to administer the bark in such quantities as the stomach would bear, either in substance, decoction, extract or tincture. This supposed specific frequently succeeded, but it also frequently failed when unassisted by other remedies. When, however, it was given at the same time with calomel and occasional cathartics it proved highly useful, especially in all those cases where visceral disease seemed to exist. When the patient underwent the slightest salivation, the paroxysms, during the mercurial action, were completely suspended, but as soon as the ptyalism began to subside, subside, so soon did the paroxysms again recur, and often with greater violence than formerly. This fact became familiar to every medical officer present.

The sulphate of copper and zinc were both frequently tried, but without the desired effect; the former sometimes, however, changed the type of the disease from quotidian or tertian to continued fever, with sickness, vomiting, and not unfrequently delirium. The latter was used in combination with the extract of cinchona as a general tonic, and in this way was found often useful after the paroxysms had subsided, and great debility remained. It was not, however, found to check the regular paroxysm, although often given with that intention. When the disease became continued, and either threatened or assumed the typhoid appearance, antimony and aromatic confection were used with success. In this case, camphor and ammonia were also very efficacious remedies. Yeast was likewise used in many desperate cases with the best effect. By this alone I have often seen patients recover when their bodies were covered with petechiæ, and when syngultus had subsisted for two or three days, and the stomach rejected every thing, even wine. Nitric acid was found very useful in almost all those cases in which mercury had been exhibited, and after the action of which the paroxysms had again returned. In these cases it not unfrequently effected a cure either alone, or given in some bitter decoction. Opium, either with or without æther, given in large doses at the approach of the fit, always shortened the cold stage, and often completely subdued the paroxysm. Venesection was also often found useful when the disease was combined with pulmonic affections, which very frequently happened. I used this remedy very often, and must confess without ever observing the least injurious consequence, but on the contrary the most speedy relief.

The objections which were most frequently urged against this most *extensive* and *potent* remedy (as Haller calls it) were that, as debility was the most prominent feature of the disease, the abstraction of blood under such circumstances appeared paradoxical. This reasoning at first appeared satisfactory in theory, but not in practice, as I was sufficiently convinced. Its salutary effects, I think, may be thus explained. The men when apparently free from disease, but in a very weak and debilitated state, were often unavoidably exposed to the vicissitudes of weather, and were very liable to be affected with the slightest change of any kind, in their languid state. This immediately produced a relapse, which in general was attended with inflammation of some of the viscera. This conclusion is sufficiently warranted from the depredations predations made on these organs, as the subsequent dissections will amply shew.

The symptoms were pyrexia, pain of the head, difficult respiration, troublesome cough, and pains of the sides or breast, but more frequently the latter. In this state I immediately used the lancet, which, in general, gave so much relief, as seldom to require a repetition. Diluents and laxatives were now continued for some days, till the inflammatory diathesis seemed subdued, then in the convalescent stage aromatics and bitters were found highly useful, and soon completely restored the enervated frame.

When lately perusing Sydenham, I found that the practice in some measure coincides with that laid down by that experienced physician in the treatment of chlorosis. In this disorder, though in general considered a disease of debility, he found that the most successful mode of treating it was, by commencing the cure with the loss of some blood, and afterwards administering tonics.

Haller, and Sir John Pringle, mention also a similar practice in intermittents—but to return. I have also tried the effects of ligatures on the extremities, with a view to check the fit, but never observed any beneficial result except in one case, where imagination and not the ligature appeared to produce the effect; the particulars of which were as follow. Going into a Ward where the experiment had not been made, and finding a man shivering horribly with gnashing teeth, I requested the Orderly to bring me a tourniquet. As soon as it entered the Ward, the patient's eyes were fixed on it, and he began, as he afterwards informed me, to wonder what I was about to do with him. I now proceeded to fix it on his arm, when the paroxysm immediately subsided, and the subsequent hot stage was very slight from the short duration of the cold. I afterwards tried it repeatedly, screwing one on opposise extremities, but without the least effect.

Epithems of nicotiana were also used with the same intention, but seldom produced any good effect, and if continued for any length of time, they did much mischief by the sickness and vomiting which they produced.

### *Treatment of the hydropic complaints.*

These were only another form or stage, and consequence of the original disease. Debility, and consequent loss of tone of the absorbent and exhalent vessels, which is the proximate cause of this disease, were the effects of the former. In this, as well as the last, a variety of remedies were had recourse to, the chief of which were squills, digitalis, submuriate of mercury, acetate of potash, nitrate of potash, and the the supertartrate of potash; elaterium, horseradish, mustard, and camboge were also frequently used with success. By the use of these separately, or in combination, the greater number were evacuated of the superabundant fluid, and the cure was completed by the administration of decoctions of quassia, serpentaria, senega, and other bitter and astringent tonics. None of these remedies operated with so much effect, or with such immediate relief to the patient, as those which possessed both a purgative and diuretic quality. Of these camboge and elaterium were the chief. They were often used with remarkable success, especially the former.

In all cases where the disease attacked suddenly, causing considerable distension of the abdomen with œdema of the legs, its exhibition was attended with the greatest success, one dose being, in general, sufficient for the evacuation of the water. It often produced vomiting, purging, and copious diuresis.

The patients thus suddenly relieved, were seldom observed to relapse into a similar state, as mentioned by authors, after the administration of these drastic hydragogues, but this might be, in part, owing to the tonics and bitters which were always exhibited after the evacuation of the water.

There were a few cases, in which the patients being nearly exhausted by the continuance of the disease, the use of these remedies was prohibited. Some of these were tapped as a dernier resource, but the fluid soon collecting again, eventually proved fatal. Others, of several months continuance, were speedily relieved by the spontaneous occurrence of a diarrhæa or dysentery, which, in general, however, proved fatal. Of this I could mention several examples, but one will suffice.—Johnes was first attacked with the fever, from which having recovered, he was discharged. Soon after he relapsed, and was now attacked with a violent pneumonia, which was, with some difficulty, subdued, by the loss of between forty and fifty ounces of blood, and other proper remedies. Being again convalescent, he was afflicted with a dropsical complaint; he swelled to an amazing size, and continued so for five or six months in despite of all our hydropic remedies. About this time he was first seized with a diarrhæa, which, by continuance, degenerated into a dysentery, and of which he died four or five days after its appearance, completely destitute of any dropsical complaint.

With respect to the dysenteric affections, which form the third most common termination of this malady; it did not appear to be contagious, but originated sporadically in different parts, and seldom or never infected those in the same Ward with the patient. It was, indeed, sometimes induced by by the accumulation of putrid animal effluvia, of which the following are sufficient examples :—

Mr. B. surgeon, was in the habit of dissecting much, and making anatomical preparations. The room in which he dissected and kept his preparations, adjoined and opened into that in which he slept. Another medical collegue who attended some of these dissections, brought his child once or twice with him to this anatomical theatre. It was seized in a day or two after with dysentery and died; the body was inspected and left no doubt of the disease. Mr. B. continuing his investigations, was soon after attacked with the same complaint, which remained very violent for several days, notwithstanding every means were used to check its progress. Soon after, finding no abatement, he was removed from this room to other lodgings, where the disease abating, he soon recovered.

These cases show that it arises, in general, more from putrid animal effluvia, than from any peculiar matter generated during the progress of the disease.

The treatment pursued in this disease was varied according to symptoms; laxatives were often given in the commencement or inflammatory stage. When it resisted these, opium and compound powder of ipecacuanha were found very efficacious; infusions of rhubarb and small doses of ipecacuanha were also useful. When the pain of the abdomen was very great, a blister applied to the hypogastric region, often relieved very desperate symptoms. In one instance, in which the complaint had continued very violent for several days, resisting every means that could be devised, the patient was seized with vomiting, which returned on the introduction of any thing into the stomach, and thus prevented the exhibition of any medicine in this way. Injections of opium and starch were often given him without any beneficial effect. In this desperate state, a large blister was applied to the lower part of the abdomen, which drawing well, he was found next morning, free from both purging and vomiting. From this time he soon recovered without any other medicine, except a few occasional doses of Dover's powder. Flannel rollers were also found useful for the support of the abdomen against that distressing symptom tenesmus.

In some instances where the disease assumed a chronic form, mercury was used, but never having witnessed its effects in such cases, I am unable to speak decisively with regard to its success.

Having thus completed my remarks with regard to the cure, I now proceed to point out the ravages the disease entailed tailed on the different organs, as demonstrated by dissection. This is an interesting part of my subject, and will, I doubt not, open a new field for the speculation of the pathologist. In this part as well as the former, I shall endeavour to point out the viscera most deranged by each form of the disease. It will be recollected that the intermittents and remittents, &c. degenerating or rather assuming the continued or typhoid type, most frequently terminated fatally. Of all the bodies which I dissected, I only find the particulars of 80 cases in my note book, 36 of which were of this form, and were, generally speaking, first in point of time, 26 hydropic and 18 dysenteric.

In these the chief organ affected was the spleen. This viscus was always enlarged, often weighing from three to five pounds. Its substance was extremely soft and easily deranged. Tubercles were often found on its surface. Inflammation and ulceration, not only of its peritoneal coat, but also of its substance, were frequently observed. These ulcers were found in all their various stages and forms, from the most minute to the almost complete exulceration of the whole lienteric mass. Of this last the following is a remarkable example.

Richard Mullan was readmitted with continued fever, which soon terminated fatally. On opening the abdomen, a large sac was found to occupy all the left hypochondriac region. It was firmly united to the stomach, diaphragm, and all the neighbouring viscera. It contained near two quarts of bloody purulent matter, in the midst of which floated the spleen quite detached, and so much ulcerated and wasted as to appear only a small mass of bloody matter without any capsule; for its proper peritoneal coat was found to have formed the basis of the sac. The lower surface of the diaphragm, which seemed to compose the superior part of this sac, was inflamed and deeply ulcerated. There were many other cases of this ulceration of the spleen, some of which being seated on its superior surface, where it comes in contact with the diaphragm, had corroded and destroyed that muscle, so as to form a communication between the thorax and abdomen, into the former of which they were found sometimes to have discharged their purulent contents.

Hard stony concretions were also frequently found on it, and in one or two instances its capsule had become cartilaginous. There was likewise an instance of accidental death from a rupture of this organ. Two of the convalescents disputing in their ward proceeded to blows, and one having fallen, his adversary was observed to have struck him while down with his foot. He rose, however, of his own accord, and ascended his birth, where he expired in a few minutes. Soon after the abdomen was found to have swelled considerably, and on inspecting the body there were found several pounds of fluid blood diffused in it. The rupture or rather laceration of the spleen was about an inch and half in length, and situated on that part of it which lies in contact with the stomach. It was also much enlarged and very soft.

The liver was the next grand organ on which this disease was found to have made rapid progress towards its destruction. Its most common appearance was preternatural enlargement, flaccidity, and induration or condensation. It was also frequently covered with coagulable lymph and adhered to the surrounding viscera. Inflammation and ulceration, not only of its coat, but also of its substance, were often detected. These were not, however, of so frequent occurrence in this organ as in the spleen. Its substance often exhibited a mottled friable appearance, with a deficiency of the secretion of bile in the biliary ducts, although the gall bladder was, in general, discovered to be quite full, and often distended with dark inspissated bile resembling tar.

Hydatids were also sometimes found in its substance. A bag of this kind was once discovered in the substance of the liver, which contained near a quart of limpid fluid. This was retained in a large sac, the structure of which appeared very white and cartilaginous. Tubercles were likewise sometimes observed in it.

The gall bladder was sometimes found in an inflamed state. In general it was distended with dark green viscid bile; sometimes, however, it was much contracted; gall-stones were often detected in it.

The stomach was for the most part found natural. It was often, however, much contracted from its middle to the pylorus. In many cases it was considerably distended with flatus. In a few instances inflammation and ulceration of its coats were discovered.

These ulcers of the stomach do not exactly resemble those of other parts of the body. In these cases there is little circumjacent inflammation. The ulcer appears of a circular form, with smooth edges, and little inflammation. At first view they appear as if cut out with an edged instrument.* In many cases the stomach was considerably corroded by the gastric juice, particularly towards its greater curvature. The peritoneum was in many cases much diseased. Its most frequent appearance was that of inflammation and adhesion. Its surface was often covered with purulent matter. Those duplicatures of the peritoneum which form the omenta, were

* Vide Baillie's Morbid Anatomy.

very very frequently nearly absorbed. In many cases nothing of it remained bu[illegible] of vessels attached to the stomach and colon, of a dark [illegible] greenish colour.

The intestines were in general natural. The most common diseased appearance was that of inflammation and consequent adhesion. Intus-susceptio was also frequently observed.

The kidneys were for the most part healthy, except in those cases of dropsy which shall be hereafter mentioned. Stones were sometimes found in the pelvis of the kidney, as well as in the ureters.

The morbid appearance which the thoracic viscera often exhibited, was also very various and considerable.

Inflammation of the pleura and lungs, with all its consequences, was, however, the most common. In some instances the surface of the lungs was thickly encrusted with a cartilaginous substance. It was very common to find the substance of the lungs anasarcous, and sometimes their structure was so far changed and condensed as to resemble the liver.

The pericardium was also sometimes found inflamed, and formed adhesions with the heart. It almost always contained a preternatural collection of serous fluid.

The heart itself did not escape the inroads of this destructive disease. Its adipose substance was often dropsical, and it was not unusual to find depositions of coaguable lymph on its surface. In one instance the surface of the right ventricle was found much inflamed and ulcerated, especially towards its superior part which approaches the auricle. On opening the ventricle the tricuspid valves were discovered to be in a complete state of ossification, by which the opening between the auricle and ventricle was nearly obliterated. The symptoms of this patient were so vicarious as to induce his medical attendants to suppose that he was affected with a cancer of the stomach.

Brain.—This intricate organ was not so frequently investigated as I could have wished, and principally for this obvious reason, that it required a greater length of time for this purpose than could be obtained. There were, however, sufficient demonstrations of the brain to show its general appearance in this disease. It was for the most part natural; there seemed, however, a great determination of blood to this organ, its vessels being in general found turgid. Adhesions of the dura mater to the surface of the brain, and depositions of lymph were often detected. A superabundant quantity of water was very often discovered in the ventricles.—In one or two cases death suddenly occurred while the patient laboured under salivation. In one of these that I inspected, a quantity of blood was effused between the brain and dura

 mater, mater, similar to the appearance observed in cases of apoplexy. The swelling of the neck and throat being so much increased as to impede the blood returning from the head, is generally supposed to be the cause of such unexpected occurrences.

I must not forget to mention that large collections of purulent matter in different parts of the body often proved fatal.

These critical abscesses were in general situated on the lumbar region, the thighs, legs, and shoulders, &c.

The matter detached the cellular substance and fascia from the muscles, insinuating itself between them, and thus formed sinuses and fistulous openings in distant and different parts. These openings were so numerous, and discharged such quantities daily, that the poor sufferers were rendered horrible spectacles. They often continued in this state for months before death terminated their wretched existence.

With respect to the morbid appearance of the hydropic form of the disease, I have little to add to the above-mentioned organic derangements. Here, however, the kidneys always seemed more flaccid and wasted than natural. The liver appeared always less or more diseased, and the whole of the peritoneum was often found to have formed various and extensive adhesions and indurations of its substance. In one case where the hydropic patient was affected with a cutaneous eruption, the whole of the peritoneum exhibited a similar appearance to that of the surface of the body.

The thorax was most frequently the seat of these complaints. Perhaps this may be explained by its being more liable to inflammatory attacks than any other cavity of the body. Adhesions of the lungs, pleura and pericardium were found in almost every instance. The substance of the lungs was very frequently anasarcous, and on pressing them, the water was discharged as from a wet sponge. The pericardium was often much distended with fluid, when no other cavity was found to contain any preternatural quantity, measuring often from two to twelve ounces. This preternatural collection of water in some of the cavities was one of the most frequent occurrences met with, under almost every form of this malady. This was not only contained in the abdominal and thoracic cavities, but was frequently found to exist in the ventricles of the brain itself.

In the dysentery, which was the third and last most common form of this disease, the morbid appearance was mostly confined to the intestines; but as the patients all laboured, in some degree, under the influence of the original complaint, the the other viscera in general, were also found to be much deranged.

The great intestines, especially from the sygmoid flexure to the anus, were always much contracted, thickened, inflamed, and ulcerated. The disease seemed to have commenced near the anus, as from thence it was observed gradually to decrease. The ulcers were so united, that the whole inner surface of the gut appeared in a state of granulation. This often extended to the cæcum, and sometimes to the smaller intestines; they commonly contained little fæcal matter, and for the most part only bloody mucus. Scybala were very seldom detected.

Having now finished these remarks, I presume no one, who will attentively peruse them, can draw any other conclusion, than that this disease was originally of an inflammatory kind. This alone can explain the direful ravages of the viscera, than which humanity never, perhaps, laboured under a greater multiplicity inflicted in so short a period.

W. HAMILTON.

*Ipswich, October 17th,* 1810.

